# The Bacterial and Fungal Microbiota of Nelore Steers Is Dynamic Across the Gastrointestinal Tract and Its Fecal-Associated Microbiota Is Correlated to Feed Efficiency

**DOI:** 10.3389/fmicb.2019.01263

**Published:** 2019-06-25

**Authors:** Déborah Romaskevis Gomes Lopes, Alex J. La Reau, Márcio de Souza Duarte, Edenio Detmann, Cláudia Braga Pereira Bento, Maria Eugênia Zerlotti Mercadante, Sarah Figueiredo Martins Bonilha, Garret Suen, Hilario Cuquetto Mantovani

**Affiliations:** ^1^Departamento de Microbiologia, Universidade Federal de Viçosa, Viçosa, Brazil; ^2^Department of Bacteriology, University of Wisconsin–Madison, Madison, WI, United States; ^3^Departamento de Zootecnia, Universidade Federal de Viçosa, Viçosa, Brazil; ^4^Instituto de Zootecnia, Centro APTA Bovinos de Corte, São Paulo, Brazil

**Keywords:** GIT microbiota, beef cattle, 16S rRNA gene, ITS1, fecal samples, residual feed intake, next-generation sequencing

## Abstract

The ruminant gastrointestinal tract (GIT) microbiome plays a major role in the health, physiology and production traits of the host. In this work, we characterized the bacterial and fungal microbiota of the rumen, small intestine (SI), cecum and feces of 27 Nelore steers using next-generation sequencing and evaluated biochemical parameters within the GIT segments. We found that only the bacterial microbiota clustered according to each GIT segment. Bacterial diversity and richness as well as volatile fatty acid concentration was lowest in the SI. Taxonomic grouping of bacterial operational taxonomic units (OTUs) revealed that *Lachnospiraceae* (24.61 ± SD 6.58%) and *Ruminococcaceae* (20.87 ± SD 4.22%) were the two most abundant taxa across the GIT. For the fungi, the family *Neocallismastigaceae* dominated in all GIT segments, with the genus *Orpinomyces* being the most abundant. Twenty-eight bacterial and six fungal OTUs were shared across all GIT segments in at least 50% of the steers. We also evaluated if the fecal-associated microbiota of steers showing negative and positive residual feed intake (n-RFI and p-RFI, respectively) was associated with their feed efficiency phenotype. Diversity indices for both bacterial and fungal fecal microbiota did not vary between the two feed efficiency groups. Differences in the fecal bacterial composition between high and low feed efficiency steers were primarily assigned to OTUs belonging to the families *Lachnospiraceae* and *Ruminococcaceae* and to the genus *Prevotella*. The fungal OTUs shared across the GIT did not vary between feed efficiency groups, but 7 and 3 OTUs were found only in steers with positive and negative RFI, respectively. These results provide further insights into the composition of the Nelore GIT microbiota, which could have implications for improving animal health and productivity. Our findings also reveal differences in fecal-associated bacterial OTUs between steers from different feed efficiency groups, suggesting that fecal sampling may represent a non-invasive strategy to link the bovine microbiota with productivity phenotypes.

## Introduction

Food-producing animals, such as monogastrics and ruminants, are responsible for most of the meat and milk produced worldwide, and these food products represent the main source of protein in the human diet. Brazil is one of the main producers of beef in the world, holding about 15% of the global meat exports (Food and Agriculture Organization of the United Nations [FAO], 2016). More than 90% of the Brazilian commercial cattle herds are animals belonging to the Nelore breed, a variety of Zebu (*Bos indicus*) that is well adapted to the environmental conditions and tropical production systems endemic to Brazil, thereby enabling production that is of global economic importance (Associação Brasileira das Indústrias Exportadoras de Carnes [ABIEC], 2017).

In order to make this production system more profitable and sustainable, farmers often seek strategies to decrease feeding costs through manipulation of rumen fermentation (e.g., ionophores) (Benatti et al., 2017; Crossland et al., 2017); altering management practices (e.g., supplements) (Jose Neto et al., 2016; Carvalho et al., 2017) and selection of cattle that have better feed conversion efficiencies (Nkrumah et al., 2006; Fidelis et al., 2017). Most of these practices target the ruminant gastrointestinal tract (GIT) microbiome and its function, as it is known to play a key role in the physiology of the mammalian host. For example, the GIT microbiota stimulates the immune system, produces vitamins and can inhibit pathogenic bacteria (Ley et al., 2006; Turnbaugh and Gordon, 2009; Cho and Blaser, 2012).

This association is even more relevant in ruminants since herbivorous animals are entirely dependent on symbiotic associations with anaerobic microorganisms from the GIT microbiota to digest plant cell wall polysaccharides (e.g., cellulose, hemicelluloses, and pectins) in the rumen (Krause and Russell, 1996). The rumen represents the main site for converting the ingested dietary components into energy to the host, while the microbiota colonizing the distal GIT (small and large intestine) are considered crucial for animal health, and to a lesser extent, the energy-harvesting functions of the host (Mao et al., 2015; Myer et al., 2015c).

Therefore, investigating the differences in microbial community composition across the ruminant GIT can provide further insights linking animal phenotypes and production traits with variation in microbial colonization and fermentation at different portions of the GIT. Previously, Oliveira et al. (2013) described the composition of the bacterial community in the GIT of a Nelore steer. However, this characterization was performed using a single animal, and changes in community composition were not linked to any specific phenotype. To gain a more in-depth understanding of the Nelore GIT microbiota, we investigated the bacterial and fungal composition of the rumen, small intestine (SI), cecum and feces of 27 Nelore steers showing differences in feed efficiency. Previous studies associating ruminant feed efficiency to microbial communities have focused primarily on the analysis of ruminal samples (McCann et al., 2014; Jewell et al., 2015; Shabat et al., 2016) whose collection depends on invasive methods or animal slaughter. Here, we investigated the compositional profile of the bacterial and fungal microbiota colonizing the Nelore GIT and also examined the hypothesis that steers with high and low feed efficiency show differences in their fecal microbiota.

Our results revealed that, despite variation in the composition of bacterial and fungal communities across different portions of the GIT, some OTUs known to be functionally relevant for fiber degradation and host development were shared across the entire GIT and present within the feces. Additionally, OTUs found in fecal samples were related to feed efficiency, suggesting that fecal microbiota analysis could be a practical method to assess differences in animal phenotypes, health status, or to monitor digestive disorders and the effect of diet, additives or supplements in cattle herds in a non-invasive manner.

## Materials and Methods

### Animals, Diets, and Sampling

The experimental procedures were approved by the Ethics Committee on Animal Use of the Instituto de Zootecnia (CEUA-IZ, Protocol 213-15), in accordance with guidelines of São Paulo State Law No. 11.977, Brazil, and by the Ethics Committee on Production Animal Use of the Universidade Federal de Viçosa (CEUAP-UFV, Protocol 026/2015).

Twenty-seven Nelore steers from the Centro Avançado de Pesquisa Tecnológica dos Agronegócios de bovinos de corte, a subsidiary of the Instituto de Zootecnia (São Paulo State, Brazil) and averaging 22.5 ± SD 0.8 mo of age and 401 ± SD 42 kg of BW were confined in individual pens (4 × 2 m) with free access to the diet and water. These animals originated from a breeding program to select steers with improved individual performance and their residual feed intake (RFI) was identified during the growth period using the GrowSafe system, as previously described (Fidelis et al., 2017). For the finishing period, the steers were adapted to the diets, facilities, and management for 22 days and remained on the finishing diet for a 103-days period. The finishing diet was composed of 333 g/kg corn silage, 17 g/kg *Brachiaria* hay, 465 g/kg dry ground corn, 163 g/kg soybean meal, 6 g/kg urea, 4 g/kg ammonium sulfate, and 13 g/kg mineral mixture (dry matter basis), formulated to meet the requirements of 1.3 kg of daily gain with a target finish weight of at least 550 kg.

After finishing, steers were transported (130 km distance) to an experimental slaughterhouse (Pirassununga, São Paulo, Brazil). Fecal samples were collected the day before the slaughter and stored in sterile plastic containers at -20°C. Animal handling was conducted in accordance with good animal welfare practices, and slaughtering procedures followed strict guidelines established and regulated by the Sanitary and Industrial Inspection Regulation for Animal Origin Products, including a fasting period of 16 h (Brasil, 2017). After slaughter, the ruminal, intestinal (mid-jejunum) and cecal contents were collected in plastic sterile containers and stored at -20°C for further analyses. Rumen samples were filtered through four layers of cheese cloth to separate the liquid from the solid fraction.

### Concentration of Volatile Fatty Acids

Organic acids were determined by HPLC using a Dionex Ultimate 3000 Dual detector HPLC (Dionex Corporation, Sunnyvale, CA, United States) coupled to a refractive index (RI) Shodex RI-101 maintained at 40°C using an ion exclusion column Phenomenex Rezex ROA, 300 × 7.8 mm maintained at 45°C. The mobile phase was prepared with 5 mmol/l sulfuric acid (H_2_SO_4_) in ultrapure water, filtered (0.22 μm) and degassed using a vacuum pump. The flow rate of the mobile phase was 0.7 ml/min. The samples (2.0 ml) were centrifuged (12,000 × *g*, 10 min) and the cell-free supernatants were treated with 600 μl of calcium hydroxide and 300 μl of cupric sulfate as described by Siegfried et al. (1984). Samples were freeze/thawn and centrifuged (12,000 × *g*, 10 min) three times before concentrated sulfuric acid (28 μl) was added to the cell-free supernatants. Stock solutions of standards were prepared using the following organic acids: acetic, succinic, propionic, valeric, isovaleric, isobutyric, and butyric acid. All organic acids were prepared with a final concentration of 10 mmol/l, except isovaleric acid (5 mmol/l) and acetic acid (20 mmol/l). Stock solutions were diluted 2-, 4-, 8-, and 16-fold in 5 mmol/l H_2_SO_4_ and used as standards in the HPLC analysis.

### DNA Extraction and Sequencing

Total genomic DNA from each sample was extracted following a mechanical disruption and phenol/chloroform extraction protocol described by Stevenson and Weimer (2007), which has been shown to generate high-quality, high-abundance DNA representative of complex bacterial community (Henderson et al., 2013). Extracted genomic DNA was quantified using a Nanodrop spectrophotometer (Thermo Scientific, Wilmington, DE, United States) and DNA was shipped for sequencing to the University of Wisconsin-Madison (United States).

The V4 hypervariable region of the bacterial 16S rRNA gene (length, ca. 250 bp) and the fungal internal transcribed spacer (ITS1; length, ca. 250 bp) were amplified using primers described by Kozich et al. (2013) and Kittelmann et al. (2013), respectively. Bacterial PCR reactions consisted of 50 ng of the total DNA, 0.4 μM of each primer, 1X Kapa Hifi HotStart ReadyMix (KAPA Biosystems), and water to 25 μl. For fungi, total DNA was increased to 100 ng and primers to 1.6 μM each. PCR was performed at 95°C for 3 min, 95°C for 30 s, 55°C for 30 s, 72°C for 30 s (25 cycles for V4 region and 35 cycles for ITS1) and a final extension step at 72°C for 5 min. PCR products were purified by PureLink^®^ Pro 96 PCR Purification Kit (Invitrogen, Carlsberg, CA, United States) and a second PCR was performed on the resulting amplicons to attach Illumina sequencing adapters and unique dual indices. PCR reactions were similar to those for V4 except that 5 μl of non-quantified PCR products were used as template DNA and 8 cycles were performed. PCR products were recovered by gel extraction in AquaPōr LM low-melt agarose (National Diagnostics, Atlanta, GA, United States) using the Zymoclean Gel DNA Recovery Kit (Zymo Research, Irvine, CA, United States). Purified DNA was quantified by Qubit^®^ Fluorometer (Invitrogen, Carlsbad, CA, United States) and equimolar amounts were pooled to create a single sample at 1 × 10^9^ ng per μl (Dias et al., 2017). Paired-end sequencing was performed using the v2 kit (2 × 250 bp) for V4 region and v3 kit (2 × 300 bp) for ITS1 on an Illumina MiSeq following manufacturer’s guidelines (Illumina, Inc., San Diego, CA, United States). All DNA sequences have been deposited in the NCBI’s Sequence Read Archive (SRA) under BioProject accession number PRJNA512996.

### Sequence Analysis

Prior to analysis, all raw sequences obtained from the Illumina MiSeq were demultiplexed using the Illumina software system in order to remove sequencing adapters and low-quality base pair calls. Bacterial and fungal sequences were then processed separately using mothur (v1.39.5) following the MiSeq SOP (Schloss et al., 2009). Briefly, paired-end reads were joined using the *make.contigs* command with default parameters, and sequences shorter than 200 bp or longer than 500 bp for Bacteria and shorter than 200 bp or longer than 600 bp for Fungi were removed. For both, sequences containing ambiguous characters or exhibiting a homopolymer run greater than 8 bp were removed. The V4 sequences were aligned using the SILVA 16S rRNA gene reference database release 128 (Quast et al., 2012) and the ITS1 sequences were aligned using the UNITE database (Kõljalg et al., 2013). Sequences that did not align to the correct location were removed. Identical sequences were grouped using the *unique.seqs* command and sequences that had two or fewer base pairs different were considered the same and grouped using *pre.cluster*. Chimeric sequences were detected using the UCHIME algorithm (Edgar et al., 2011) and removed. Singletons (sequences that occurred only once in the entire dataset) were also removed. Bacterial and fungal sequences were taxonomically assigned using *classify.seqs* to the SILVA and UNITE gene reference databases, respectively, with a bootstrap cut-off of 80. The method of Naive Bayesian Classifier (NBC) was used to find the taxonomy of query sequences (Wang et al., 2007). Bacterial and fungal sequences were grouped into operational taxonomic units (OTUs) using uncorrected pairwise distances clustered with the furthest neighbor method and the average neighbor method, respectively, with a similarity cut-off of 97%. Before proceeding with analysis, the coverage of all samples was assessed by Good’s coverage. Due to different sequencing depths, OTU tables were normalized to equal sequence counts (5,122 sequences for Bacteria and 1,707 for Fungi, established by the sample that presented the lowest number of sequences). The normalized OTU tables were used to determine alpha diversity indices (Chao1, Shannon and Simpson) and to calculate relative abundances of OTUs.

### Statistical Analysis

Differences in bacterial and fungal alpha-diversity, as well as in VFA concentration across the GIT were performed in MiniTab^®^ 17.1.0 (Minitab, Inc., Quality Plaza, 1829 Pine Hall Road, State College, Pennsylvania 16801, United States) by ANOVA, followed by *post hoc* Tukey test. *P*-values below 0.05 were considered significant. To evaluate the clustering of steers using OTU composition across the GIT, a non-metric multidimensional scaling (nMDS) analysis using the Bray-Curtis dissimilarity index (beta diversity index), and non-parametric analyses of similarities (ANOSIM, number of permutation = 10,000) were performed using the Past software package (Hammer et al., 2001).

Venn diagrams were constructed using the tools available in the “Bioinformatics and Evolutionary Genomics” website to visualize shared and exclusive OTUs^1^. These analyses were performed using OTUs that were present in at least 50% (up to 100%, according to the OTU being analized) of all steers analyzed in this study (≥13 steers). This criteria was based on previous observations indicating that species of ruminal bacteria that exhibit a heritable component show high presence (≥50%) across animals (Sasson et al., 2017).

Non-metric multidimensional scaling analysis was used to represent the beta-diversity of bacterial and fungal communities of fecal samples from steers showing positive (p-RFI, low feed efficiency) and negative residual feed intake (n-RFI, high feed efficiency). To compare bacterial and fungal composition of the fecal samples from steers exhibiting either p-RFI or n-RFI, Venn diagrams were constructed using the OTUs that were present in at least 50% (up to 100%) of the steers belonging to each feed efficiency group (≥7 animals to the p-RFI group and ≥6 animals to the n-RFI group). The Kolmogorov-Smirnov, Shapiro-Wilk and D’Algostino and Pearson tests were performed using GraphPad Prism v. 5.00 (GraphPad Software, San Diego, CA, United States) to determine if the relative abundance of bacterial and fungal OTUs followed a Gaussian distribution. Since most OTUs did not follow this distribution (*P* > 0.05), the differences in relative abundances according to feed efficiency group were assessed by White’s non-parametric *t*-test using the software package STAMP v 2.1.3 (Parks et al., 2014). *P*-values below 0.05 were considered significant. The bacterial shared OTUs were plotted on a volcano plot to visualize the differences in relative abundances of OTUs present in the n-RFI steers versus the p-RFI steers. To accomplish this, the fold change (ratio between n-RFI and p-RFI relative abundance of each OTU) was assessed along with the *p*-Values obtained using White’s non-parametric *t*-test to visualize the data. Adjustments for multiple comparisons were not performed due to the exploratory nature of this study. In addition, differences in alpha-diversity of fecal samples according to each feed efficiency group were assessed by *t*-tests in MiniTab^®^ 17.1.0 (Minitab, Inc., Quality Plaza, 1829 Pine Hall Road, State College, Pennsylvania 16801, United States). *P*-values below 0.05 were considered significant.

## Results

### Sequencing

In total, we generated 10,000,278 raw bacterial sequences with an average length of 253 bp across all samples. For fungi, we generated 7,226,285 raw sequences with an average length of 241 bp and across all samples. After trimming, quality filtering and removal of chimeras, 5,697,607 (mean 42,204 ± SD 45,615 to samples) and 3,676,969 (mean 28,068 ± SD 19,009 to samples) high-quality bacterial and fungal sequences were obtained, respectively. Good’s coverage across samples was >97% for bacterial sequencing and >90% for fungal sequencing, indicating that our sequencing efforts sufficiently covered the diversity of bacterial and fungal communities in rumen liquids (RL) and solids (RS), SI, cecum and feces. The summary of sequence counts and OTUs that passed the steps of filtering, clean up and normalization are shown in Supplementary Table S1. Only reads and OTUs present in at least 50% of all the steers in a specific RFI group (≥7 animals in p-RFI and ≥6 animals in n-RFI) were considered for analysis of the feed efficiency phenotype (^∗^cut-off in Supplementary Table S1).

### The Bacterial Microbiota Changes Across the Gastrointestinal Tract

**FIGURE 1:**
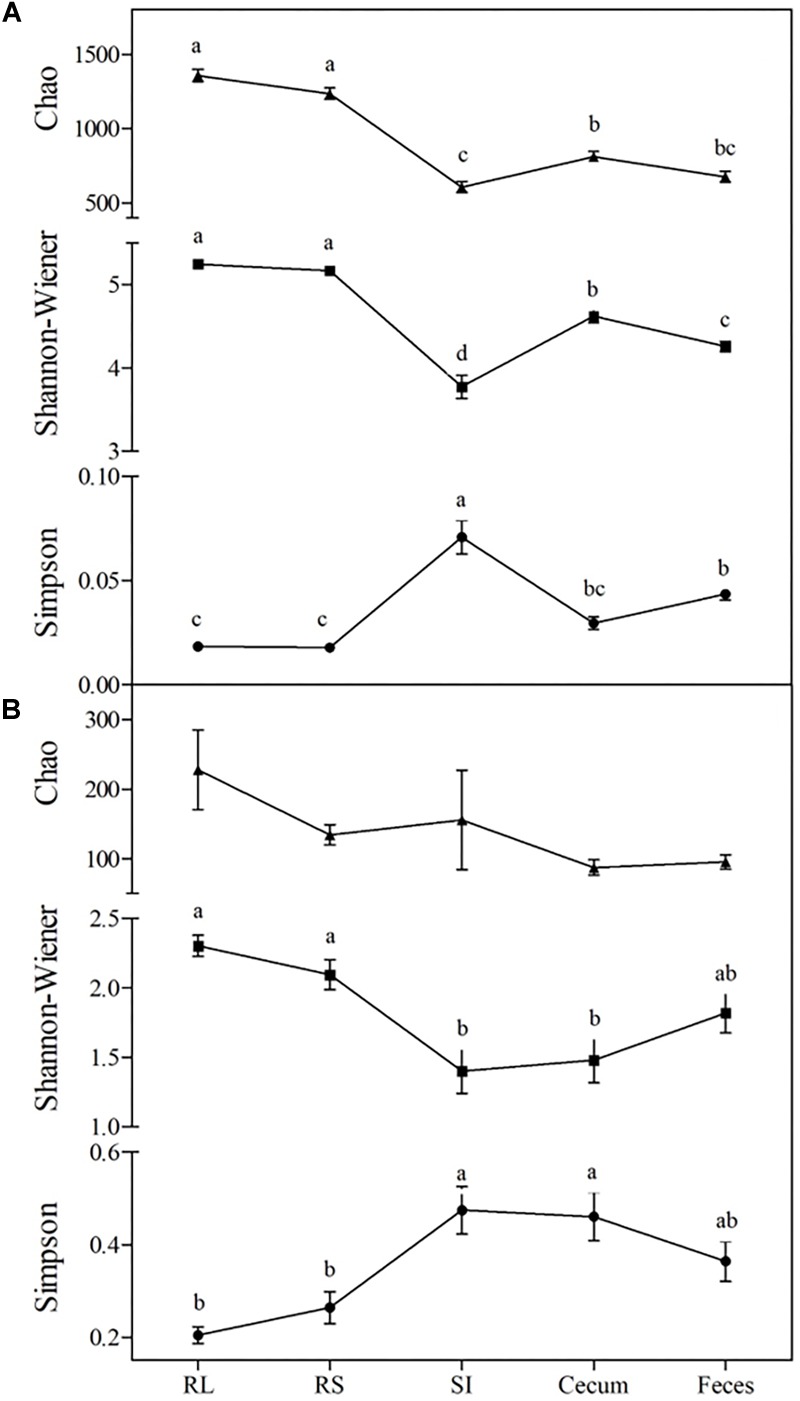
Changes in alpha-diversity of bacterial **(A)** and fungal **(B)** communities across the GIT of Nelore steers. Triangles represent Chao richness, squares represent Shannon-Wiener diversity and circles represent Simpson’s diversity index. For each index, means followed by at least one same letter did not differ at a 5% level of significance as determined by Tukey’s test. RL, rumen liquid; RS, rumen solids, and SI, small intestine.

The intra-community diversity (alpha diversity) varied across different portions of the GIT (Tukey’s test, *P* < 0.05). Maximum values of Chao richness and Shannon diversity were observed in the RL and RS communities (1,296 ± SD 207 and 5.21 ± SD 0.26, respectively), while minimal values of Chao richness (641 ± SD 189) and Shannon’s diversity (3.78 ± SD 0.73) were found in the SI. The Simpson diversity index showed the inverse trend, with maximum values observed in the SI community (0.071 ± SD 0.042) and minimal values in the RL, RS, and cecal communities (0.022 ± SD 0.01) (Figure 1A).

**FIGURE 2:**
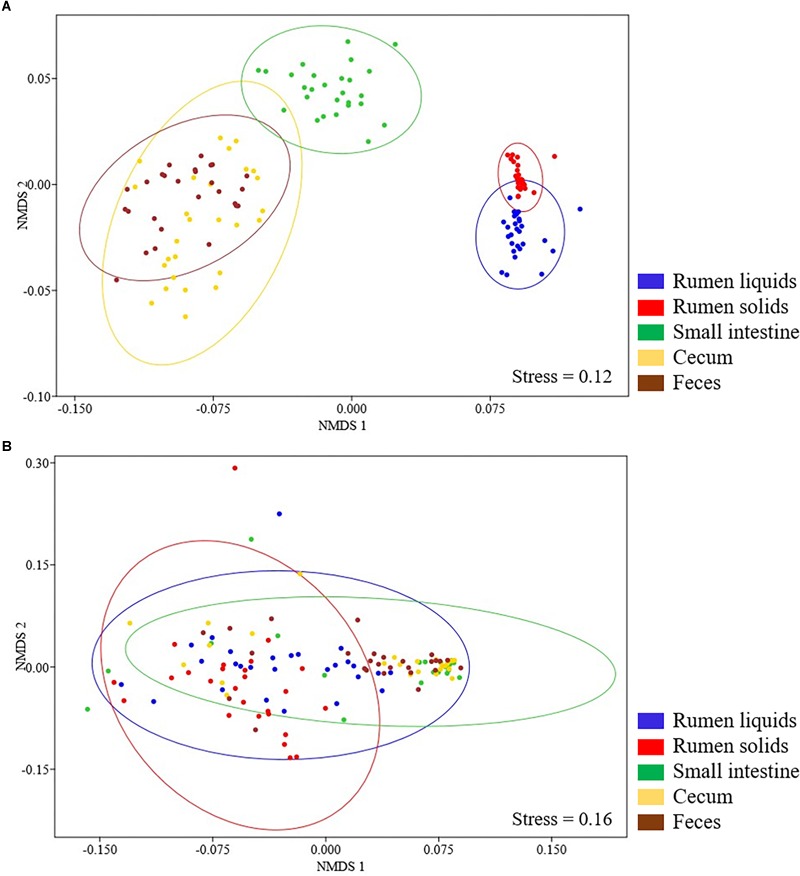
Non-metric multidimensional scaling (nMDS) plots of the Bray-Curtis dissimilarity index for bacterial **(A)** and fungal **(B)** communities in the GIT of Nelore steers. Individual points represent GIT samples from different steers and different colors represent distinct GIT segments. Ellipses represent 95% confidence intervals.

Beta diversity analysis showed that the Bray-Curtis dissimilarities of the bacterial communities differed according to GIT segment (ANOSIM, *P* < 0.001). Main differences were observed comprising the rumen (RL and RS fractions), the SI and the large intestine (cecum and feces), and the bacterial communities in the RL/RS and cecum/feces samples also differed from each other (ANOSIM, *P* < 0.001) (Figure 2A).

**FIGURE 3:**
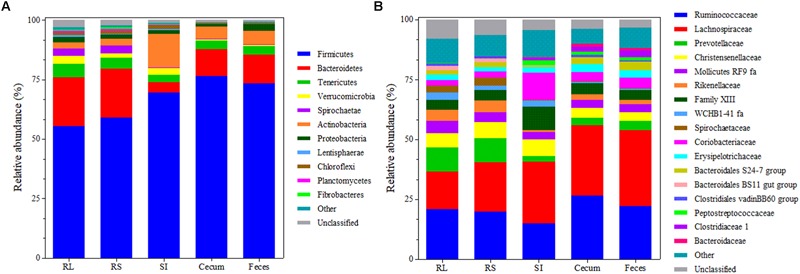
Bacterial composition at the phylum **(A)** and family **(B)** level across the GIT of Nelore steers. Each bar represents the mean bacterial community composition in rumen liquids (RL), rumen solids (RS), small intestine (SI), and cecum and feces. For phylum, “other” corresponds to the sum of phyla that showed relative abundance <0.5%. For family, “other” corresponds to the sum of families that showed relative abundance <1%.

Taxonomic analysis of the GIT bacterial communities revealed 5,230 unique OTUs (mean 551 ± SD 188 per sample after normalization) that were assigned to 24 phyla, 50 classes, 84 orders, 163 families, and 402 genera. The unclassified group represented 1.66 (± SD 1.01%), 2.48 (± SD 1.26%), 2.91 (± SD1.34%), 5.19 (± SD 1.89%), and 27.65 (± SD 5.18%) of the OTUs that could not be assigned to any phylum, class, order, family or genus, respectively. *Firmicutes* represented the predominant phylum in the bacterial community across the GIT of Nelore steers (relative abundance 66.68 ± SD 9.15%), followed by *Bacteroidetes* in RL, RS, cecum and feces samples (relative abundance of 20.50 ± SD 1.53%, 20.57 ± SD 1.59, 11.34 ± SD 1.55, and 12.17 ± SD 1.74, respectively) and members of the phylum *Actinobacteria* in SI samples (13.90 ± SD 2.05%) (Figure 3A).

The most represented families across the GIT included the *Lachnospiraceae* (24.61 ± SD 6.58%) and *Ruminococcaceae* (20.87 ± SD 4.22%). *Prevotelaceae* was also predominant in ruminal bacterial communities (10.06 ± SD 2.25%), but its abundance decreased (*P* < 0.05) in other portions of the GIT (3.15 ± SD 0.76%) (Figure 3B).

**FIGURE 4:**
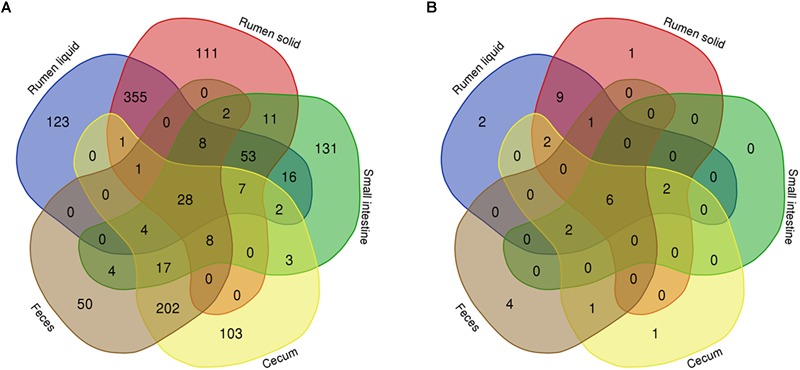
Venn diagrams showing the number of bacterial **(A)** and fungal **(B)** OTUs shared between rumen (liquid and solids), small intestine, and cecum and feces. Only bacterial OTUs that were present in at least 50% of all steers (13 animals) from each GIT segment are represented.

To filter the differences between the bacterial communities across the GIT, we used Venn diagrams to analyze OTUs from each portion of the GIT that were present in at least half of the Nelore steers (≥13 steers for each portion of the GIT). We found 1,241 OTUs that were distributed across all samples, 9.91% which were exclusive to RL, 8.94% to RS, 10.56% to SI, 8.3% to cecum, and 4.03% to the feces (Figure 4A).

**FIGURE 5:**
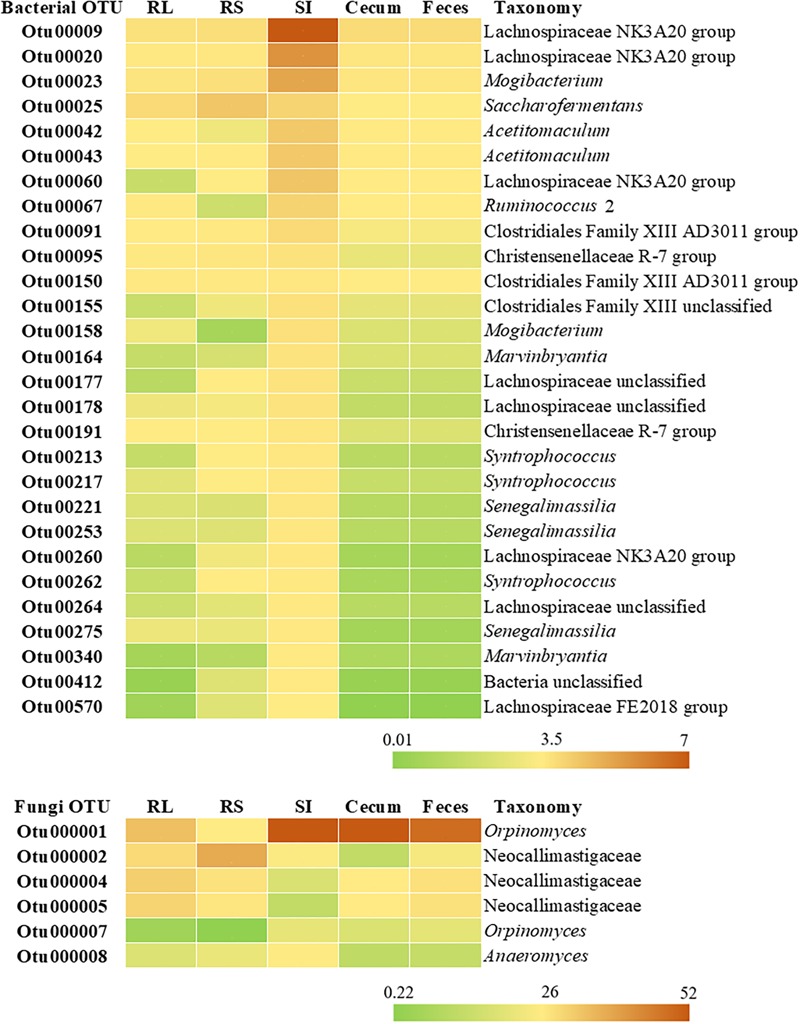
Heatmap representing the relative abundance (%) of bacterial and fungal OTUs shared between rumen liquid (RL), rumen solids (RS), small intestine (SI), and cecum and feces. The taxonomy for each OTU is given at the highest classifiable level.

Taxonomic classification of the 28 OTUs shared across all 5 segments of the GIT showed that these sequences belonged to the *Clostridiales* (24 OTUs) and *Coriobacteriales* order (3 OTUs). One OTU shared by all GIT portions could not be assigned to any phylum. The highest classifiable level for the shared OTUs, as well the relative abundance across the GIT were represented in a heatmap (Figure 5). The majority of the shared OTUs showed higher abundances in the bacterial community colonizing the SI (with some OTUs also being more abundant in the RS community), but decreased in the large intestine with similar abundances in the cecum and feces. The most abundant OTUs in the SI were assigned to the order *Clostridiales*, with OTU00005 (genus *Romboutsia*, 10.51%), OTU00007 (genus *Paeniclostridium*, 8.93%), OTU00003 (genus *Peptostreptococcaceae unclassified*, 7.21%), OTU00008 (genus *Peptostreptococcaceae unclassified*, 7.15%), and OTU00009 (genus *Lachnospiraceae NK3A20 group*, 6.61%) being the most representative ones.

### The Fungal Ruminal Microbiota Differs From the Distal Segment of the Gastrointestinal Tract

The Shannon and Simpson indexes of the fungal community varied between the rumen and the distal (SI and cecum) segments of the GIT (Tukey’s test, *P* < 0.05). The maximum values of Shannon diversity and minimum values of Simpson’s index were observed in the RL, RS, and fecal communities (2.07 ± SD 0.61 and 0.28 ± SD 0.18, respectively). The fungal chao richness did not vary across the GIT (140 ± SD 211, Tukey’s test, *P* > 0.05) (Figure 1B). The fungal beta diversity, summarized on a nMDS plot with Bray-Curtis dissimilarities, showed that the rumen fungal communities differed (ANOSIM, *P* < 0.001) between the liquid and solid fractions (RS and RL). In addition, the RS and RL fungal communities were also distinct from those in distal segments of the GIT (SI, cecum and fecal samples) (ANOSIM, *P* < 0.001). However, it should be pointed out that no differences were observed between the fungal communities in the SI, cecum and fecal samples (ANOSIM, *P* > 0.001) (Figure 2B).

Taxonomic analysis of the GIT fungal communities revealed 3,253 unique OTUs (mean 62 ± SD 34 per sample after normalization) that were assigned to 3 phyla, 6 classes, 6 orders, 8 families, and 12 genera. The unclassified group represented 13.47 (± SD 10.28 %), 13.77 (± SD 10.44 %), 13.77 (± SD 10.44 %), 15.65 (± SD 13.89 %) and 68.77 (± SD 18.33 %) of the OTUs that could not be assigned to any phylum, class, order, family or genus, respectively. The OTUs assigned to the *Neocallimastigaceae* family represented more than 75% of the fungal OTUs across all segments of the GIT (Table 1).

**Table 1:** Fungal composition profile across the GIT of Nelore steers.

Level	Taxa	Rumen liquids	Rumen solids	Small intestine	Cecum	Feces
Phylum	Neocallimastigomycota	85.51 ± 11.88	95.04 ± 4.98	86.71 ± 13.23	85.77 ± 9.76	77.29 ± 12.35
	Others	0.32 ± 0.36	0.07 ± 0.09	0.47 ± 1.91	0.29 ± 0.37	1.16 ± 1.69
	Unclassified	14.17 ± 11.59	4.89 ± 4.91	12.82 ± 13.23	13.94 ± 9.56	21.55 ± 12.12
Class	Neocallimastigomycetes	85.51 ± 11.88	95.04 ± 4.98	86.71 ± 13.72	85.77 ± 9.76	77.29 ± 12.35
	Others	0.00 ± 0.00	0.00 ± 0.00	0.39 ± 1.83	0.05 ± 0.22	0.41 ± 1.76
	Unclassified	14.49 ± 11.878	4.96 ± 4.98	12.90 ± 13.23	14.18 ± 9.75	22.30 ± 12.36
Order	Neocallimastigales	85.51 ± 11.88	95.04 ± 4.98	86.71 ± 13.72	85.77 ± 9.76	77.29 ± 12.35
	Others	0.00 ± 0.00	0.00 ± 0.00	0.38 ± 1.80	0.05 ± 0.22	0.38 ± 1.72
	Unclassified	14.49 ± 11.88	4.96 ± 4.98	12.91 ± 13.23	14.18 ± 9.75	22.33 ± 12.36
Family	Neocallimastigaceae	83.22 ± 15.35	91.72 ± 14.07	84.88 ± 15.13	84.46 ± 12.55	76.61 ± 12.39
	Others	0.00 ± 0.00	0.00 ± 0.00	0.38 ± 1.80	0.05 ± 0.25	0.38 ± 1.75
	Unclassified	16.78 ± 15.44	8.28 ± 14.10	14.74 ± 14.85	15.49 ± 12.61	23.01 ± 12.45
Genera	*Anaeromyces*	2.31 ± 1.76	1.65 ± 0.63	1.51 ± 1.17	0.98 ± 0.81	1.21 ± 1.12
	*Cyllamyces*	0.34 ± 1.11	0.85 ± 3.04	1.52 ± 6.46	0.69 ± 2.93	0.29 ± 1.30
	*Neocallimastix*	1.53 ± 1.02	1.42 ± 0.74	0.69 ± 0.69	0.42 ± 0.39	0.65 ± 0.61
	*Orpinomyces*	13.38 ± 12.58	3.51 ± 3.24	44.15 ± 29.85	41.70 ± 27.90	36.18 ± 22.09
	Others	0.11 ± 0.19	0.12 ± 0.16	0.45 ± 1.94	0.10 ± 0.35	0.40 ± 1.80
	Unclassified	82.33 ± 12.18	92.45 ± 4.73	51.68 ± 27.14	56.11 ± 26.48	61.27 ± 21.14

*The values represent the mean of relative abundance and standard deviation (%). “Others” corresponds to the sum of taxa that showed relative abundance <1% in all TGI portions.*

Considering only the 31 fungal OTUs that were present in at least half of the Nelore steers (≥13 steers), our Venn diagrams showed that 6.15% were exclusive to the RL, 3.23% to the RS, none to the SI, 3.23% to the cecum and 12.9% to the feces (Figure 4B). The six shared OTUs distributed between all five segments of the GIT of Nelore steers belonged to the *Neocallimastigaceae* (Figure 5).

### Microbial Fermentation Profile Changes Across the Gastrointestinal Tract

Analysis of the microbial fermentation profiles across the GIT segments showed the highest concentration (65.13 mmol/l) of total volatile fatty acids (VFAs) in the cecum and feces, while the SI showed total VFA concentration almost six times lower (11.57 mmol/l) than other GIT segments (ANOVA, *P* < 0.05) (Table 2). In the rumen, cecum and feces, the proportions of acetic, propionic and butyric acids were greater than other VFAs (*P* < 0.05). In the SI samples, succinic acid was present at a high proportion (23.88%), in addition to acetic and propionic acids (Table 2).

**Table 2:** Fermentation profiles of Nelore steers GIT portions.

	Rumen		Small intestine		Cecum		Feces	
Parameter	Mean	SEM	Mean	SEM	Mean	SEM	Mean	SEM
Acetic acid(%)	69.36a	0.37	55.52b	2.99	65.95a	1.29	69.39a	0.85
Propionic acid(%)	14.51a	0.38	16.71a	2.41	15.65a	0.75	13.17a	0.35
Butyric acid(%)	6.88c	0.19	0.81d	0.29	9.01b	0.56	12.46a	0.62
Isobutyric acid(%)	3.77ab	0.17	3.08b	0.70	5.82a	0.98	2.11b	0.18
Valeric acid(%)	0.97b	0.04	–	–	1.96a	0.16	1.96a	0.16
Isovaleric acid(%)	3.86a	0.19	–	–	1.60b	0.18	0.61c	0.07
Succinic acid(%)	0.65b	0.07	23.88a	2.00	–	–	0.31b	0.11
A/P ratio^∗^	4.87ab	0.14	8.21a	1.80	4.49b	0.24	5.40ab	0.19
Total VFa ^∗∗^ (mmol/l)	51.80b	3.15	11.57c	1.28	66.96a	3.19	63.30a	3.25

*The values represent the mean and standard error of mean (SEM). To each parameter, means followed by at least one same letter did not differ at the 5% level of significance by the Tukey’s test. ^∗^A/P, Acetic to propionic acid ratio; ^∗∗^, Total concentration of volatile fatty acids (VFAs); (-), not detected.*

### Specific Bacterial and Fungal OTUs in Fecal Samples Are Associated With the p-RFI and n-RFI Phenotype in Nelore Steers

For the 27 steers used in this study, previous feed efficiency analysis performed during the growth period (approximately 12 months prior to sampling) identified 12 steers with n-RFI and 15 steers with positive RFI (p-RFI). The RFI values were significantly different (*P* < 0.05) between n-RFI (-0.93 ± SD 0.17) and p-RFI steers (0.87 ± SD 0.14), separating the animals into two groups: high and low feed efficiency, respectively. OTU analyses were conducted using fecal samples from these two feed efficiency groups to address the hypothesis that the fecal microbiome of n-RFI and p-RFI Nelore steers show differences in their bacterial and fungal community composition.

Alpha diversity analysis of the bacterial and fungal communities in the fecal samples showed that Chao1 richness, Simpson’s diversity, and Shannon’s diversity did not vary significantly between RFI groups (*t*-test *P* > 0.05) (Supplementary Table S2). Analysis of beta diversity in fecal samples did not show differences (ANOSIM, *P* > 0.001) between bacterial and fungal communities of p-RFI and n-RFI steers (Supplementary Figure S1). However, a more detailed analysis of the OTUs present in at least half of the steers classified in each feed efficiency group (≥7 animals to the p-RFI group and ≥6 animals to the n-RFI group) using Venn diagrams showed bacterial and fungal OTUs that were unique to each efficiency group.

**FIGURE 6:**
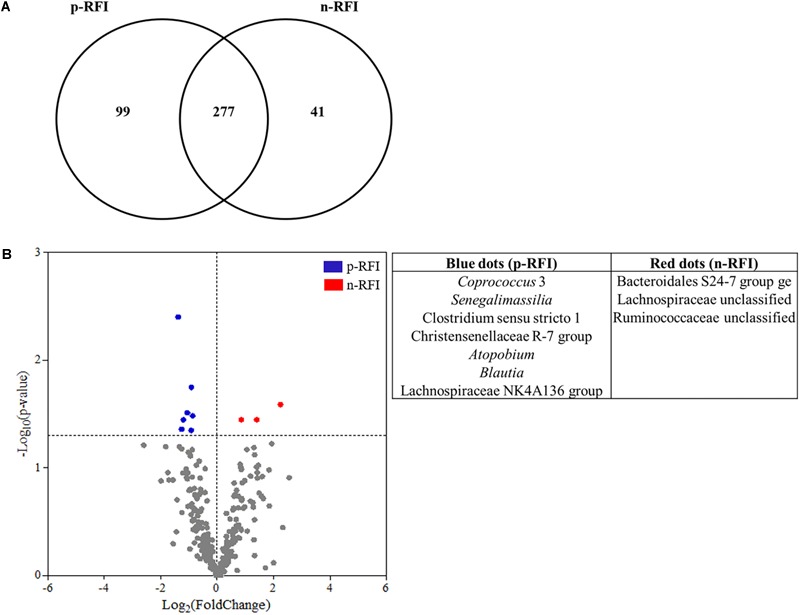
Differences in the fecal bacterial community from p-RFI and n-RFI Nelore steers. **(A)** Venn diagram showing the number of bacterial OTUs shared in the fecal samples of p-RFI and n-RFI Nelore steers. Only bacterial OTUs that were present in at least 50% of the steers from each feed efficiency group (at least 7 animals to p-RFI and 6 animals to n-RFI) are represented. **(B)** Differences in the relative abundance of shared OTUs from feces of Nelore steers. Each point represents an OTU and points that showed Log_2_(FoldChange) > 0 were OTUs showing higher abundance in n-RFI steers, while points that showed Log_2_(FoldChange) < 0 were OTUs showing higher abundance in p-RFI steers. Red points are OTUs statistically more abundant in n-RFI steers and blue points represent OTUs that were statistically more abundant in p-RFI steers (White’s non-parametric *t*-test, *P* < 0.05).

**FIGURE 7:**
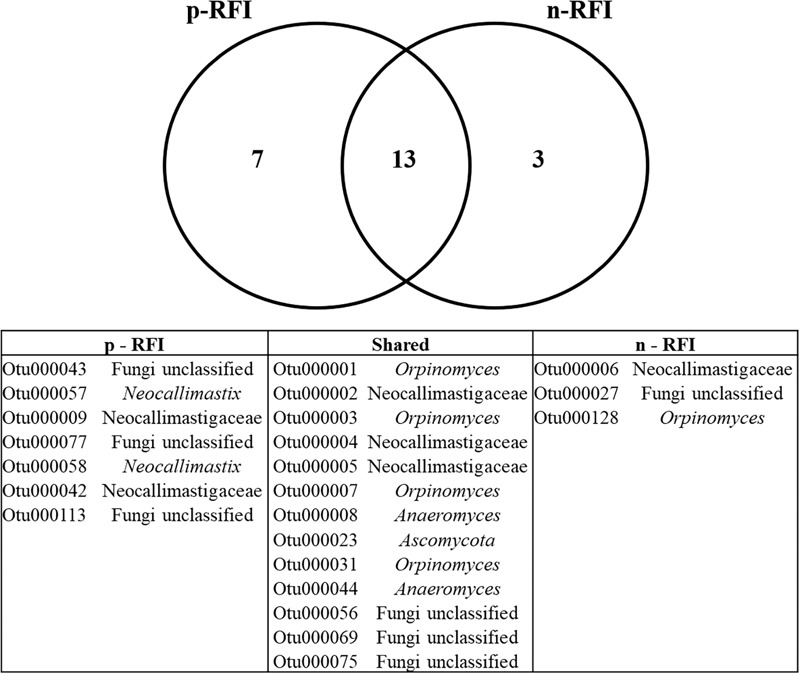
Venn diagram showing the number of fungal OTUs shared in the feces of p-RFI and n-RFI Nelore steers. Only fungal OTUs that were present in at least 50% of the steers from each feed efficiency group (7 animals in p-RFI and 6 animals in n-RFI) are represented.

For the bacteria, 99 OTUs were unique to the p-RFI steers (corresponding to 5.16% of the total relative abundance), while the n-RFI steers had 41 unique OTUs representing 1.54% of total relative abundance (Figure 6A). The most abundant bacterial OTUs present only in p-RFI or n-RFI steers were OTU0034 (*Alloprevotella*) and OTU00234 (*Turicibacter*), respectively (Supplementary Table S3). Differences in relative abundance of the bacterial OTUs shared between the p-RFI and n-RFI groups (66.43% of the total OTUs) were represented on a volcano plot (Figure 6B). Of the 277 shared OTUs, 46.57% showed higher abundance in the n-RFI steers with only 3 OTUs significantly more abundant in these steers (White’s non-parametric *t*-test, *P* < 0.05). For the p-RFI steers, 53.43% of the OTUs showed higher abundance in these steers, relative to the n-RFI group and only seven OTUs were found to be significantly more abundant (White’s non-parametric *t*-test, *P* < 0.05) (Figure 6B). Among the OTUs that were statistically more abundant in n-RFI steers, OTU00065 (unclassified *Lachnospiraceae*) represented almost 1% of the relative abundance of OTUs in this group. In the p-RFI steers, OTU00153 (*Coprococcus*) had relative abundances 2.5 times greater in p-RFI than in n-RFI steers (Supplementary Table S4). For fungi, seven OTUs were unique to the fecal microbiome of p-RFI steers (corresponding to 4.55% of relative abundance), while the n-RFI steers had three unique OTUS representing 1.14% of total relative abundance (Figure 7 and Supplementary Table S5). The majority of these unique fecal-associated OTUs were assigned to the family *Neocallimastigaceae*. The relative sequence abundance of shared fungal OTUs were not different between the two efficiency groups (White’s non-parametric *t*-test, *P* > 0.05, Supplementary Table S6).

## Discussion

The GIT of animals is differentiated anatomically into defined and adapted regions (stomach, SI and large intestine) that are colonized by microorganisms capable of metabolizing ingested dietary substrates (Turnbaugh and Gordon, 2009). Ruminants have forestomachs (rumen, reticulum, and omasum) that are responsible for the digestion and conversion of most dietary components into VFAs that represent the major source of energy for ruminants (Russell and Hespell, 1981; Jami and Mizrahi, 2012). Because the rumen is the primary site of feed fermentation, most studies evaluating the ruminant microbiome have focused on the ruminal ecosystem (Tajima et al., 2001; Pitta et al., 2010; Li et al., 2012; Firkins and Yu, 2015; Shabat et al., 2016; Li and Guan, 2017). However, studies have demonstrated relevant associations between the GIT microbiota and ruminant production (Myer et al., 2015b,c, 2016, Lindholm-Perry et al., 2016). Although it has been shown that different portions of the GIT harbor distinct microbial communities with different biochemical properties, relevant species of microorganisms are known to be shared across all GIT regions (Frey et al., 2009; Oliveira et al., 2013).

In this study, we characterized the bacterial and fungal composition of the GIT of 27 Nelore steers, an important beef cattle breed due to its prominence in world meat production. Our data revealed significant differences in the bacterial and fungal community (beta diversity) between the evaluated segments (Figure 2) and supports the idea that passage of digesta through adjacent GIT sections affects the composition of the gut microbial communities (Oliveira et al., 2013; Mao et al., 2015; Perea et al., 2017). Mao et al. (2015) characterized the bacterial communities colonizing different regions within the GIT of Holstein dairy cattle and reported significant differences in species composition and metabolic function of digesta-associated and mucosa-adherent microbiota. Heterogeneity in microbiota composition of distinct GIT segments have been reported not only for ruminants (Dias et al., 2017; Perea et al., 2017), but also for other animals like chickens (Clavijo and Vives Flórez, 2017) and mice (Montealegre et al., 2016).

The diversity and abundance of gut microorganisms also varies considerably according to host development and anatomical location, mainly because of varying physiochemical conditions (e.g., pH, redox potential, oxygen availability), availability of nutrients and sites for adhesion, host secretions (mucins), and exposure to exogenous compounds that cause disturbance in the ecosystem (e.g., antibiotics, dietary changes, and pathogens) (Carbonero et al., 2014; Moya and Ferrer, 2016). In our study, several differences were observed in composition, richness (Chao index), diversity (Shannon index), and species dominance (Simpson) during the passage from the rumen to the SI. The genera *Romboutsia* and an unclassified *Peptostreptococcaceae* represented the most abundant OTUs in the SI, but they were not present in other regions of the Nelore GIT. Members of the *Lachnospiraceae* NK3A20 group (OTUs OTU00009 and OTU000020) were also abundant in the SI and were also present in the rumen, cecum and fecal samples.

These differences in the microbial community across the Nelore GIT are possibly related to drastic changes in chemical (acidification and host enzymes secretion) and physical (osmolarity, adhesion sites) conditions in the abomasum (Figure 1). The secretion of enzymes by the host in the glandular stomach (abomasum) and the SI allows for digestion of microbial biomass, which provides protein to the host (Russell and Hespell, 1981). In addition, feed digestion in the SI is shorter than in the rumen due to a faster passage rate, which limits the establishment and adaptation of the microbiota to the physical and chemical conditions present in this portion of the GIT (Frey et al., 2009; Carbonero et al., 2014). This is reflected in the SI, which had the lowest total VFA concentration of all GIT segments analyzed in this study (Table 1). Microbial fermentation appears to be reestablished in the large intestine, with the proportion of acetate, propionate and butyrate being similar to the rumen (Table 2), which agrees with our observations that microbial diversity increases in the distal segments of the GIT (Figure 1).

Despite the divergences observed among the microbial communities of the GIT segments, the bacterial families *Lachnospiraceae* and *Ruminococcaceae* (Figure 3) and the fungal family *Neocallimastigaceae* (Table 1) were the most abundant across the GIT. The *Lachnospiraceae* includes species of bacteria with fibrolytic and proteolytic properties, such as *Lachnospira multiparus* and *Butyrivibrio fibrisolvens*, while members of the *Ruminococcaceae* family include cellulolytic bacteria, such as *Ruminococcus albus* and *R. flavefaciens* (Russell and Rychlik, 2001). The *Neocallimastigaceae* family comprises several anaerobic fungal species (e.g., *Orpinomyces, Anaeromyces, Neocallimastix*, and *Piromyces*) and recent transcriptomic and comparative genomics studies indicated that these species produce highly active enzymes for the degradation of plant cell walls and recalcitrant fiber in the rumen (Haitjema et al., 2017; Gruninger et al., 2018). Considering the functional role of these microbial groups in the degradation of plant biomass, their relative abundance in the GIT segments may represents mechanisms for additional energy acquisition by the host through the digestion of structural carbohydrates that escape ruminal degradation (Oliveira et al., 2013; Myer et al., 2015c). Moreover, our finding that 28 bacterial and 6 fungal OTUs were common to all segments of the GIT (Figure 5) suggest that some bacterial and fungal species are able to colonize and/or survive in all portions of the GIT. All shared bacterial OTUs represented Gram-positives and were found in greater abundances in the SI. The digestive conditions and processes that occur in the transition from the abomasum to the SI could function as a filter that limits the growth of ruminal microorganisms throughout the entire ruminant GIT (Oliveira et al., 2013; Moya and Ferrer, 2016; Myer et al., 2016). In the absence of the enzymatic and chemical processes that occur in the abomasum and the SI, the composition of microbial communities would probably be much less divergent across the GIT.

Sampling GIT contents often relies upon invasive techniques or sacrificing the animal, and some studies have suggested the use of fecal samples as a proxy to explore potential associations between the GIT microbiota and host phenotype (Turnbaugh and Gordon, 2009; Tajima et al., 2012; Nash et al., 2017; Tang et al., 2018). Here, we evaluated the relationship between fecal microbiota composition and feed efficiency and found that differences between bacterial communities of negative (n-RFI) and positive (p-RFI) feed efficiencient steers are due to OTUs belonging to the families *Lachnospiraceae* (*Blautia, Coprococcus, Butyrivibrio*, and *Roseburia* genera), *Prevotelaceae* (*Alloprevotella* and *Prevotella* genera), *Coriobacteriaceae* (*Atopodium* and *Senegalimassilia* genera), and *Ruminococcaceae* (*Saccharofermentans* genus) (Supplementary Tables S3, S4). These bacteria are typically involved in the degradation of dietary substrates and VFA production in ruminants (Russell and Rychlik, 2001; Jami and Mizrahi, 2012; Li and Guan, 2017). For example, species within the *Prevotella* and *Butyrivibrio* are well known for producing hydrolytic enzymes, such as cellulases, xylanases, and beta-glucanases, that degrade plant structural polysaccharides (Cotta and Hespell, 1986; Kamra, 2005). Furthermore, *Butyrivibrio* is involved in butyrate production, which represents one of the main sources of energy for enterocytes and exerts strong effects on GIT epithelial cells, such as stimulation of cell proliferation and differentiation (Wächtershäuser and Stein, 2000; Guilloteau et al., 2010). Butyrate also has an effect on the expression of leptin in bovine adipocytes, affecting feed intake and energy expenditure of the host (Soliman et al., 2007). In addition, ruminal abundance of members of the *Lachnospiraceae* and *Ruminococcaceae* families, as well as the genus *Prevotella*, have been reported as being associated with host feed efficiency (Carberry et al., 2012; McCann et al., 2014; Myer et al., 2015a; Shabat et al., 2016). For the fungal communities, shared OTUs did not vary in abundance between the p-RFI and n-RFI groups (Supplementary Table S6), with only a few unique OTUs identified for each group. Studies on anaerobic fungi are challenging due to their fastidious nature and information about their potential role in feed efficiency is lacking (Haitjema et al., 2017; Gruninger et al., 2018).

The results presented here expand our knowledge about the composition of the Nelore GIT microbiome and suggests specific taxa potentially associated with feed efficiency. However, it should be noted that these findings are observational, precluding inferences on causality. We observed differences between microbial communities colonizing adjacent sections of the Nelore GIT and showed that fecal samples, although distinct in composition from other GIT sections, harbor considerable microbial diversity associated with different fermentation products and feed efficiency phenotypes. This is promising, as collection of fecal samples is non-invasive and more practical for sampling large numbers of animals to study different host traits. For example, analysis of fecal samples has been applied to population-scale studies of humans, making it possible to associate changes in the GIT microbiota with the health status of individuals and several physiological and psychological conditions, including obesity, autism, humor, kidney stone, biogeography, and eating habits (Turnbaugh and Gordon, 2009; Lloyd-Price et al., 2017; Nash et al., 2017; Tang et al., 2018).

Thus, while additional studies are needed, these findings suggest that analysis of microbial communities in fecal samples could be useful for monitoring functional groups related to feed efficiency in cattle. Further confirmatory studies using larger cohorts will be needed to corroborate our findings and to evaluate the consistency in the relationship of fecal microbial populations with animal phenotype. Large-scale studies assessing the fecal microbial community of cattle herds could enable the identification of microbial groups potentially associated with a causal effect on ruminant feed efficiency. Expanding knowledge of the effects of the microbial community on the well-being and health of cattle will also be important for the development of new management, nutrition and manipulation strategies of the GIT microbial community.

## Ethics Statement

The experimental procedures were approved by the Ethics Committee on Animal Use of the Instituto de Zootecnia (CEUA-IZ, Protocol 213-15), in accordance with guidelines of São Paulo State Law No. 11.977, Brazil, and by the Ethics Committee on Production Animal Use of the Universidade Federal de Viçosa (CEUAP-UFV, Protocol 026/2015).

## Author Contributions

DL, GS, ED, and HM, conceived and designed the experiments. GS, MM, SB, ED, MSD, and HM provided experimental and laboratorial resources. DL, MSD, and HM conducted sample collection and DNA extraction. DL, CB, and AL performed library construction and sequencing. DL, AL, GS, and HM conducted data analyses and interpretation of results. DL and HM wrote the manuscript. AL, GS, MSD, and ED reviewed and edited the manuscript. All authors read and approved the final manuscript.

## Conflict of Interest Statement

The authors declare that the research was conducted in the absence of any commercial or financial relationships that could be construed as a potential conflict of interest.

## Footnotes

1 http://bioinformatics.psb.ugent.be/webtools/Venn/

## Supplementary Material

FIGURE S1 Non-metric multidimensional scaling (nMDS) plots of the Bray-Curtis dissimilarity index for bacterial **(A)** and fungal **(B)** communities in fecal samples of Nelore steers. Individual points represent fecal samples from different steers, with the green and purple dots representing the n-RFI and the p-RFI steers, respectively.

TABLE S1 Summary of sequencing data derived from GIT contents of Nelore steers.

TABLE S2 Alpha-diversity metrics of the bacterial community in fecal samples of Nelore steers according to RFI group.

TABLE S3 Most abundant exclusive bacterial OTUs identified in fecal samples of Nelore steers showing low (p-RFI) or high (n-RFI) feed efficiency.

TABLE S4 Shared bacterial OTUs that were more abundant (White’s non-parametric *t*-test, P < 0.05) in fecal samples of Nelore steers showing n-RFI (gray lines) or p-RFI (white lines).

TABLE S5 Fungal OTUs that were unique to the fecal-associated microbiome of Nelore steers showing low (p-RFI) or high (n-RFI) feed efficiency.

TABLE S6 Shared fungal OTUs from the fecal microbiome of Nelore steers showing n-RFI and p-RFI.
