# Supplementary figures and images for: The Bacterial and Fungal Microbiota of Nelore Steers Is Dynamic Across the Gastrointestinal Tract and Its Fecal-Associated Microbiota Is Correlated to Feed Efficiency
Source: Front Microbiol. 2019 Jun 25;10:1263. doi: 10.3389/fmicb.2019.01263 (PMC6603086; doi:10.3389/fmicb.2019.01263)

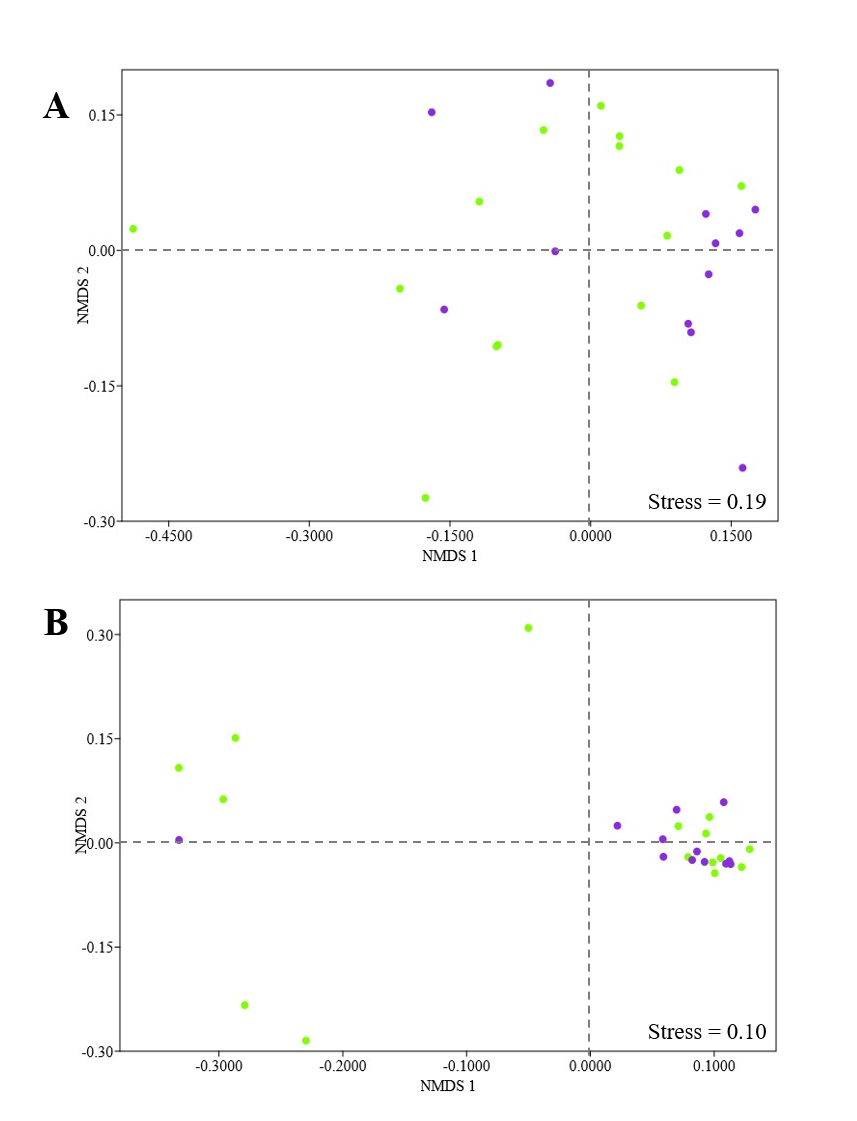

Supplement: FIGURE S1 — Non-metric multidimensional scaling (nMDS) plots of the Bray-Curtis dissimilarity index for bacterial (A) and fungal (B) communities in fecal samples of Nelore steers. Individual points represent fecal samples from different steers, with the green and purple dots representing the n-RFI and the p-RFI steers, respectively. [file Image_1.TIF]
