# Supplementary material for: The Bacterial and Fungal Microbiota of Nelore Steers Is Dynamic Across the Gastrointestinal Tract and Its Fecal-Associated Microbiota Is Correlated to Feed Efficiency
Source: Front Microbiol. 2019 Jun 25;10:1263. doi: 10.3389/fmicb.2019.01263 (PMC6603086; doi:10.3389/fmicb.2019.01263)
Supplement: TABLE S1 — Summary of sequencing data derived from GIT contents of Nelore steers. [file Table_1.docx]

**Table S1.** Summary of sequencing data derived from GIT contents of Nelore steers.

|  |  |  |  | **After filtering and clean-up** | | **After normalization** | | **After cut off*** | |
| --- | --- | --- | --- | --- | --- | --- | --- | --- | --- |
|  |  | **Steers (n)** | **Good's coverage** | **Reads** | **OTUs** | **Reads** | **OTUs** | **Reads** | **OTUs** |
| **Bacteria** | **Rumen liquid** | 27 | 0.990 ± 0.003 | 35213 ± 11737 | 1442 ± 166 | 5009 ± 44 | 773 ± 76 | 4091 ± 225 | 442 ± 35 |
|  | **Rumen solid** | 27 | 0.990 ± 0.004 | 30028 ± 8941 | 1247 ± 168 | 5034 ± 75 | 715 ± 75 | 4247 ± 256 | 451 ± 35 |
|  | **Small intestine** | 27 | 0.992 ± 0.005 | 28242 ± 14295 | 625 ± 121 | 5076 ± 34 | 362 ± 110 | 4958 ± 223 | 222 ± 36 |
|  | **Cecum** | 27 | 0.995 ± 0.002 | 50138 ± 42872 | 939 ± 279 | 5058 ± 35 | 491 ± 107 | 4312 ± 420 | 263 ± 38 |
|  | **Feces** | 27 | 0.995 ± 0.003 | 67401 ± 85626 | 907 ± 281 | 5052 ± 26 | 413 ± 82 | 4535 ± 270 | 238 ± 24 |
| **Fungi** | **Rumen liquid** | 27 | 0.949 ± 0.017 | 17840 ± 12263 | 967 ± 549 | 1611 ± 40 | 84 ± 41 | 1301 ± 322 | 17 ± 3 |
|  | **Rumen solid** | 26 | 0.948 ± 0.020 | 21640 ± 10743 | 1219 ± 370 | 1587 ± 39 | 67 ± 21 | 1244 ± 337 | 16 ± 4 |
|  | **Small intestine** | 24 | 0.956 ± 0.020 | 31594 ± 22684 | 1285 ± 682 | 1621 ± 39 | 48 ± 37 | 1259 ± 489 | 7 ± 2 |
|  | **Cecum** | 27 | 0.966 ± 0.012 | 39908 ± 25556 | 1439 ± 698 | 1625 ± 25 | 50 ± 30 | 1296 ± 439 | 10 ± 2 |
|  | **Feces** | 27 | 0.960 ± 0.008 | 29514 ± 11309 | 1331 ± 447 | 1619 ± 17 | 59 ± 29 | 1286 ± 283 | 9 ± 2 |

Values represent mean and (±) standard deviation. *Reads and OTUs that were detected in each GIT portion of at least half of the steers (≥13 animals).

**Table S2.** Alpha-diversity metrics of the bacterial community in fecal samples of Nelore steers according to RFI group.

|  | **Index** | **p-RFI** | **n-RFI** | ***P*-value** |
| --- | --- | --- | --- | --- |
| **Bacteria** | **Chao** | 710 ± 188 | 632 ± 181 | 0.213 |
|  | **Shannon** | 4.31 ± 0.28 | 4.2 ± 0.36 | 0.421 |
|  | **Simpson** | 0.04 ± 0.01 | 0.04 ± 0.02 | 0.981 |
| **Fungi** | **Chao** | 83 ± 38 | 110 ± 68 | 0.251 |
|  | **Shannon** | 1.91 ± 0.70 | 1.70 ± 0.81 | 0.480 |
|  | **Simpson** | 0.33 ± 0.22 | 0.44 ± 0,24 | 0.335 |

Values represent mean and (±) standard deviation.

**Table S3.** Most abundant exclusive bacterial OTUs identified in fecal samples of Nelore steers showing low (p-RFI) or high (n-RFI) feed efficiency.

|  |  | **Taxonomy** | **Relative abundance (%)** | **SEM** |
| --- | --- | --- | --- | --- |
| **p-RFI** | Otu00034 | *Alloprevotella* | 0.640 | 0.243 |
|  | Otu00053 | *Ruminococcaceae* UCG-005 | 0.569 | 0.250 |
|  | Otu00068 | *Bifidobacteriaceae* uncultured | 0.335 | 0.291 |
|  | Otu00033 | *Erysipelotrichaceae* UCG-003 | 0.325 | 0.256 |
|  | Otu00209 | *Bacteroidales* unclassified | 0.224 | 0.096 |
|  | Otu00476 | *Clostridium* sensu stricto 1 | 0.120 | 0.068 |
|  | Otu00382 | *Bacteroides* | 0.113 | 0.069 |
|  | Otu00129 | *Blautia* | 0.097 | 0.041 |
|  | Otu00579 | *Erysipelatoclostridium* | 0.089 | 0.033 |
|  | Otu00364 | *Lachnospiraceae* unclassified | 0.089 | 0.065 |
| **n-RFI** | Otu00234 | *Turicibacter* | 0.234 | 0.110 |
|  | Otu00706 | *Christensenellaceae* R-7 group | 0.101 | 0.053 |
|  | Otu00341 | *Butyrivibrio* 2 | 0.087 | 0.037 |
|  | Otu00142 | *Saccharofermentans* | 0.079 | 0.062 |
|  | Otu00600 | *Anaerovibrio* | 0.073 | 0.052 |
|  | Otu00222 | *Alloprevotella* | 0.071 | 0.021 |
|  | Otu00897 | *Roseburia* | 0.056 | 0.027 |
|  | Otu01035 | *Prevotella* 1 | 0.051 | 0.018 |
|  | Otu00790 | *Erysipelotrichaceae* UCG-003 | 0.046 | 0.021 |
|  | Otu00427 | *Lachnospiraceae* unclassified | 0.046 | 0.021 |

(*) Taxonomy for each OTU is given at the highest classifiable level. SEM, standard error of mean.

**Table S4.** Shared bacterial OTUs that were more abundant (White’s non-parametric t-test, *P* < 0.05) in fecal samples of Nelore steers showing n-RFI (grey lines) or p-RFI (white lines).

| **OTU** | **p-RFI** | **SEM** | **n-RFI** | **SEM** | **Taxonomy** |
| --- | --- | --- | --- | --- | --- |
| Otu00895 | 0.017 | 0.006 | 0.081 | 0.029 | *Bacteroidales* S24-7 group ge |
| Otu00065 | 0.487 | 0.087 | 0.880 | 0.135 | *Lachnospiraceae* unclassified |
| Otu00883 | 0.013 | 0.004 | 0.035 | 0.009 | *Ruminococcaceae* unclassified |
| Otu00153 | 0.240 | 0.047 | 0.092 | 0.017 | *Coprococcus* 3 |
| Otu00253 | 0.044 | 0.007 | 0.023 | 0.005 | *Senegalimassilia* |
| Otu00359 | 0.113 | 0.023 | 0.055 | 0.005 | *Clostridium* sensu stricto 1 |
| Otu00089 | 0.220 | 0.037 | 0.121 | 0.019 | *Christensenellaceae* R-7 group |
| Otu00441 | 0.030 | 0.007 | 0.013 | 0.004 | *Atopobium* |
| Otu00703 | 0.032 | 0.008 | 0.013 | 0.003 | *Blautia* |
| Otu00545 | 0.069 | 0.014 | 0.036 | 0.007 | *Lachnospiraceae* NK4A136 group |

The values represent relative abundance (%) and the standard error of mean (SEM). (*) Taxonomy for each OTU is given at the highest classifiable level.

**Table S5.** Fungal OTUs that were unique to the fecal-associated microbiome of Nelore steers showing low (p-RFI) or high (n-RFI) feed efficiency.

|  |  | **Taxonomy** | **Relative abundance (%)** | **SEM** |
| --- | --- | --- | --- | --- |
| **p-RFI** | Otu000043 | Fungi unclassified | 0.666 | 0.316 |
|  | Otu000057 | *Neocallimastix* | 0.082 | 0.030 |
|  | Otu000009 | *Neocallimastigaceae* | 3.324 | 2.385 |
|  | Otu000077 | Fungi unclassified | 0.132 | 0.071 |
|  | Otu000058 | *Neocallimastix* | 0.138 | 0.058 |
|  | Otu000042 | *Neocallimastigaceae* | 0.108 | 0.041 |
|  | Otu000113 | Fungi unclassified | 0.104 | 0.037 |
| **n-RFI** | Otu000006 | *Neocallimastigaceae* | 0.868 | 0.266 |
|  | Otu000027 | Fungi unclassified | 0.237 | 0.096 |
|  | Otu000128 | *Orpinomyces* | 0.036 | 0.011 |

(*) Taxonomy for each OTU is given at the highest classifiable level. SEM, standard error of mean.

**Table S6.** Shared fungal OTUs from the fecal microbiome of Nelore steers showing n-RFI and p-RFI.

| **OTU** | **p-RFI** | **SEM** | **n-RFI** | **SEM** | **p-value** | **Taxonomy** |
| --- | --- | --- | --- | --- | --- | --- |
| Otu000001 | 39.602 | 7.876 | 54.172 | 7.508 | 0.206 | *Orpinomyces* |
| Otu000002 | 4.861 | 2.009 | 2.288 | 0.798 | 0.305 | *Neocallimastigaceae* |
| Otu000003 | 4.383 | 2.310 | 7.441 | 4.064 | 0.586 | *Orpinomyces* |
| Otu000004 | 7.471 | 2.079 | 8.213 | 1.840 | 0.811 | *Neocallimastigaceae* |
| Otu000005 | 8.181 | 2.646 | 6.848 | 1.575 | 0.721 | *Neocallimastigaceae* |
| Otu000007 | 3.620 | 1.264 | 2.464 | 0.976 | 0.474 | *Orpinomyces* |
| Otu000008 | 3.309 | 1.535 | 0.445 | 0.150 | 0.058 | *Anaeromyces* |
| Otu000023 | 1.547 | 0.811 | 1.753 | 0.737 | 0.859 | *Ascomycota* |
| Otu000031 | 0.234 | 0.084 | 0.165 | 0.040 | 0.509 | *Orpinomyces* |
| Otu000044 | 0.483 | 0.292 | 0.150 | 0.047 | 0.368 | *Anaeromyces* |
| Otu000056 | 0.399 | 0.148 | 0.146 | 0.093 | 0.178 | Fungi unclassified |
| Otu000069 | 0.198 | 0.054 | 0.159 | 0.064 | 0.640 | Fungi unclassified |
| Otu000075 | 0.228 | 0.097 | 0.159 | 0.062 | 0.597 | Fungi unclassified |

(*) Taxonomy for each OTU is given at the highest classifiable level. SEM, standard error of mean.
